# Lysine‐241 Has a Role in Coupling 2OG Turnover with Substrate Oxidation During KDM4‐Catalysed Histone Demethylation

**DOI:** 10.1002/cbic.201800002

**Published:** 2018-04-06

**Authors:** Rebecca L. Hancock, Martine I. Abboud, Tristan J. Smart, Emily Flashman, Akane Kawamura, Christopher J. Schofield, Richard J. Hopkinson

**Affiliations:** ^1^ Department of Chemistry University of Oxford Chemistry Research Laboratory 12 Mansfield Road Oxford OX1 3TA UK; ^2^ Leicester Institute of Chemical and Structural Biology University of Leicester Henry Wellcome Building Lancaster Road Leicester LE1 7RH UK

**Keywords:** epigenetics, histone demethylase, methyllysine, oxygen sensing, transcription

## Abstract

The JmjC histone lysyl demethylases (KDMs) play important roles in modulating histone methylation states and have the potential to be regulated by oxygen availability. Lys241 of the KDM4 subfamily is proposed to be important in oxygen binding by KDM4A. We report evidence that, although Lys241 is unlikely to be directly involved in oxygen binding, it has an important role in coupling 2‐oxoglutarate cosubstrate oxidation with lysine demethylase activity. The results suggest that compounds promoting the uncoupling of substrate oxidation are of interest as JmjC‐KDM inhibitors.

Histone lysyl demethylases (KDMs) regulate gene transcription by catalysing the demethylation of methylated lysine residues on the N‐terminal tails of histones.^1^ Although the first KDMs to be identified, KDM1A/B, are amine oxidases,^2^ the majority of KDMs, that is, the human KDM2–7 subfamilies, are Jumonji‐C (JmjC) domain‐containing 2‐oxoglutarate (2OG) and iron(II)‐dependent dioxygenases (JmjC‐KDMs).^3^ JmjC‐KDMs couple the hydroxylation of methyllysine residues to the oxidative decarboxylation of 2OG, producing succinate and CO_2_; the resultant hydroxylated lysyl N‐methyl group is an unstable hemiaminal that fragments to give the demethylated lysine and formaldehyde (Scheme 1).^4^

**Scheme 1:**
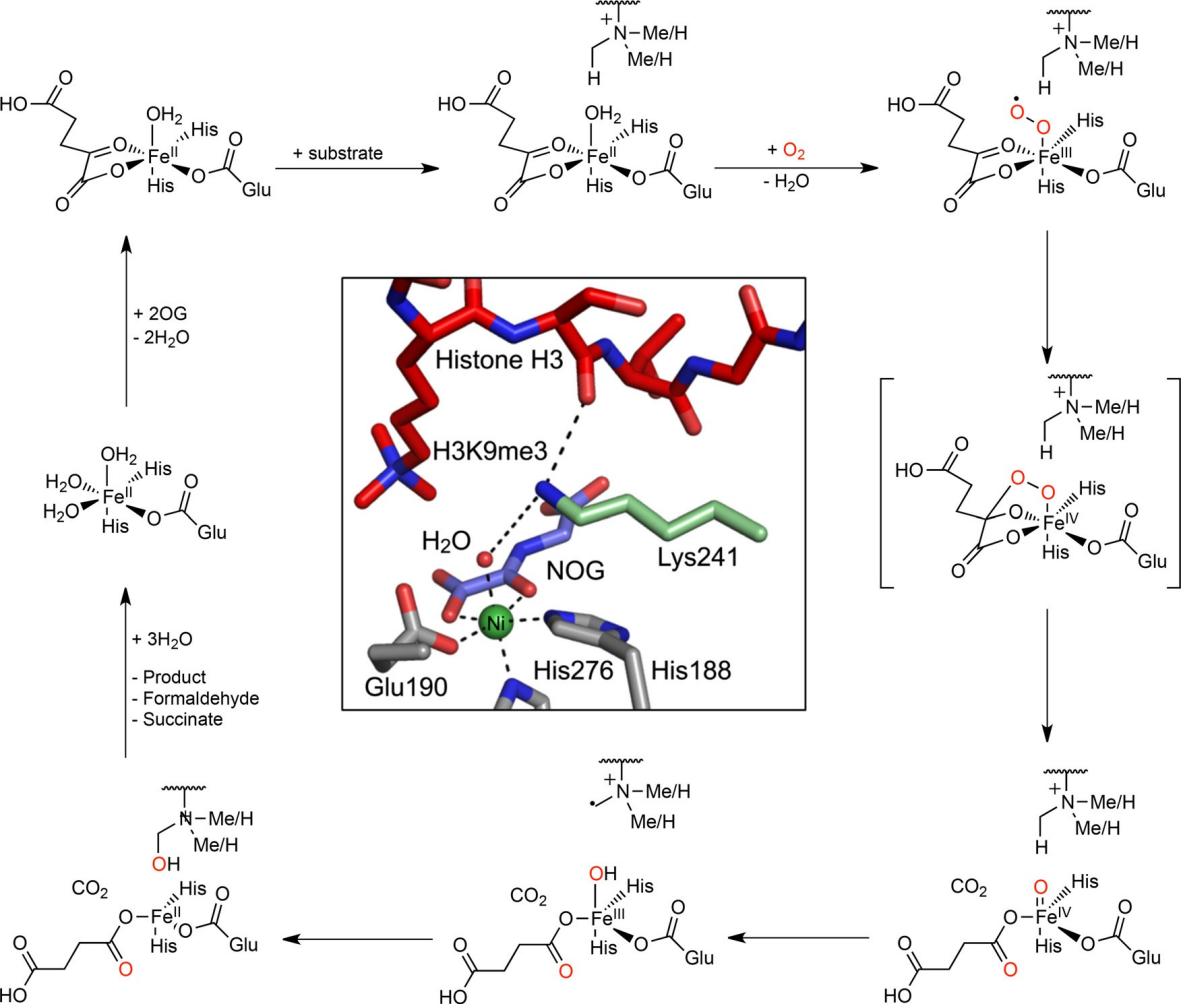
Outline mechanism of KDM4A‐catalysed demethylation. After binding of 2OG to the ferrous iron at the active site, the histone substrate binds. O_2_ then coordinates to the iron, thereby initiating an oxidative decarboxylation reaction, forming succinate, CO_2_ and a reactive iron(IV)‐oxo intermediate. Insertion of the iron(IV)‐bound oxygen atom into a histone methyl C−H bond occurs, with resultant reduction of the iron(IV) to iron(II). The hemiaminal product on the histone then fragments, giving the demethylated product and formaldehyde. Inset. A view of an X‐ray crystal structure of KDM4A complexed with Ni (substituting for Fe^II^, green)), *N*‐oxalylglycine (NOG; a 2OG analogue, blue) and a histone H3 fragment peptide *N* ^*ϵ*^‐trimethylated at Lys9 (red). Lys241 is shown in pale green (PDB ID: 2OQ6).

Despite their structural and mechanistic similarities, the KDM2–7 enzymes have diverse sequences and methylation state specificities.^5^ KDM4A–E are perhaps the best studied of the JmjC‐KDMs, and accept mono‐, di‐ and trimethylated lysines as substrates; KDM4 catalysis has been observed at H3K9, H3K36 (KDM4A–C) and H1.4K26 (KDM4A–D).^6^ KDM4A/E have also been reported to catalyse the demethylation of N‐methylated arginines in the context of histone fragment peptides.^7^

Abnormal expression patterns of JmjC‐KDMs have been linked to multiple diseases and disorders including in autism,^8^ cardiac hypertrophy,^9^ mid‐line defects^10^ and several cancers.^11^ Consequently, modulating KDM activity is of medicinal interest. However, therapeutic targeting of KDMs is currently compromised by an incomplete understanding of their biochemical and physiological roles. For example, it is possible that KDM catalysis is regulated by the availability of O_2_ within some cells, although evidence for the physiological relevance of such regulation is incomplete.^12^

X‐ray crystal structures of KDM4 enzymes in complex with substrate peptides have been reported and provided insights into mechanism and a basis for inhibition studies.^1^ An early crystallographic and mutagenesis study on KDM4A by Chen et al. identified a catalytically essential active‐site lysine residue (K241) that was proposed to regulate O_2_ binding (Scheme 1);^13^ hence K241 is potentially involved in regulating the O_2_ sensitivity of KDM4s. K241, which is conserved within the KDM4 subfamily but absent in other KDMs, is therefore an attractive target residue for KDM4‐selective inhibitors.

Here, we report biochemical studies assessing the role of K241 in KDM4A catalysis. The results reveal that the K241A KDM4A variant is capable of catalysing substrate‐uncoupled oxidative decarboxylation of 2OG at comparable efficiency to wild‐type KDM4A, thus implying a similarly efficient reaction with O_2_ (at least for uncoupled turnover). Demethylation of a trimethylated H3K9 peptide by K241A KDM4A was also observed, although the catalysis was markedly less efficient than for wild‐type KDM4A, and demethylation activity was not increased upon increasing the O_2_ concentration. Binding analyses by fluorescence polarisation indicate similar binding efficiencies of the histone substrate to both the WT enzyme and the K241A variant. Overall, the results imply that K241 is important in KDM4A catalysis; although it does have a role in coupling 2OG oxidation and demethylation, it is unlikely to be involved in regulating O_2_ binding.

Initially, we produced two recombinant variants of KDM4A (residues 1–359) in *Escherichia coli* BL21(DE3) cells; a truncated wild‐type sequence (KDM4A WT), or a variant with an alanine in place of Lys241 (K241A KDM4A). Studies were then carried out to evaluate the ability of the two KDM4A proteins to catalyse the demethylation of a histone peptide substrate. The two proteins (1 μm) were individually incubated with 2OG (100 μm, a concentration well above its WT *K*_M_ value of 26±7 μm),12b ascorbate (100 μm), ferrous iron (10 μm) and a 15‐residue H3K9me3 peptide (100 μm, sequence: ARTKQTARKme3STGGKA) in HEPES buffer (50 mm, pH 7.5) at 37 °C; the extent of the reaction was determined by using MALDI‐TOF mass spectrometry after 40 min. Substantial demethylation of the H3K9me3 peptide was observed in the sample with WT KDM4A, resulting in formation of both di‐ and monomethylated products (H3K9me2 and H3K9me1 respectively, Figure 1 A). The K241A variant, however, manifested only minimal KDM activity; no H3K9me1 or unmethylated peptide (H3K9me0) was evident, although low levels of H3K9me2 were observed (Figure 1 A). Demethylation was not increased by increasing the concentration of 2OG to 2 mm (Figures S1 and S2 in the Supporting Information). These findings suggest that K241 is required for efficient KDM activity, thus supporting previous work that also employed MS.^13^ Minimal K241A KDM4A‐catalysed demethylation activity was observed with other histone fragment peptides reported to be KDM4A substrates (Figure 1 B).

**Figure 1:**
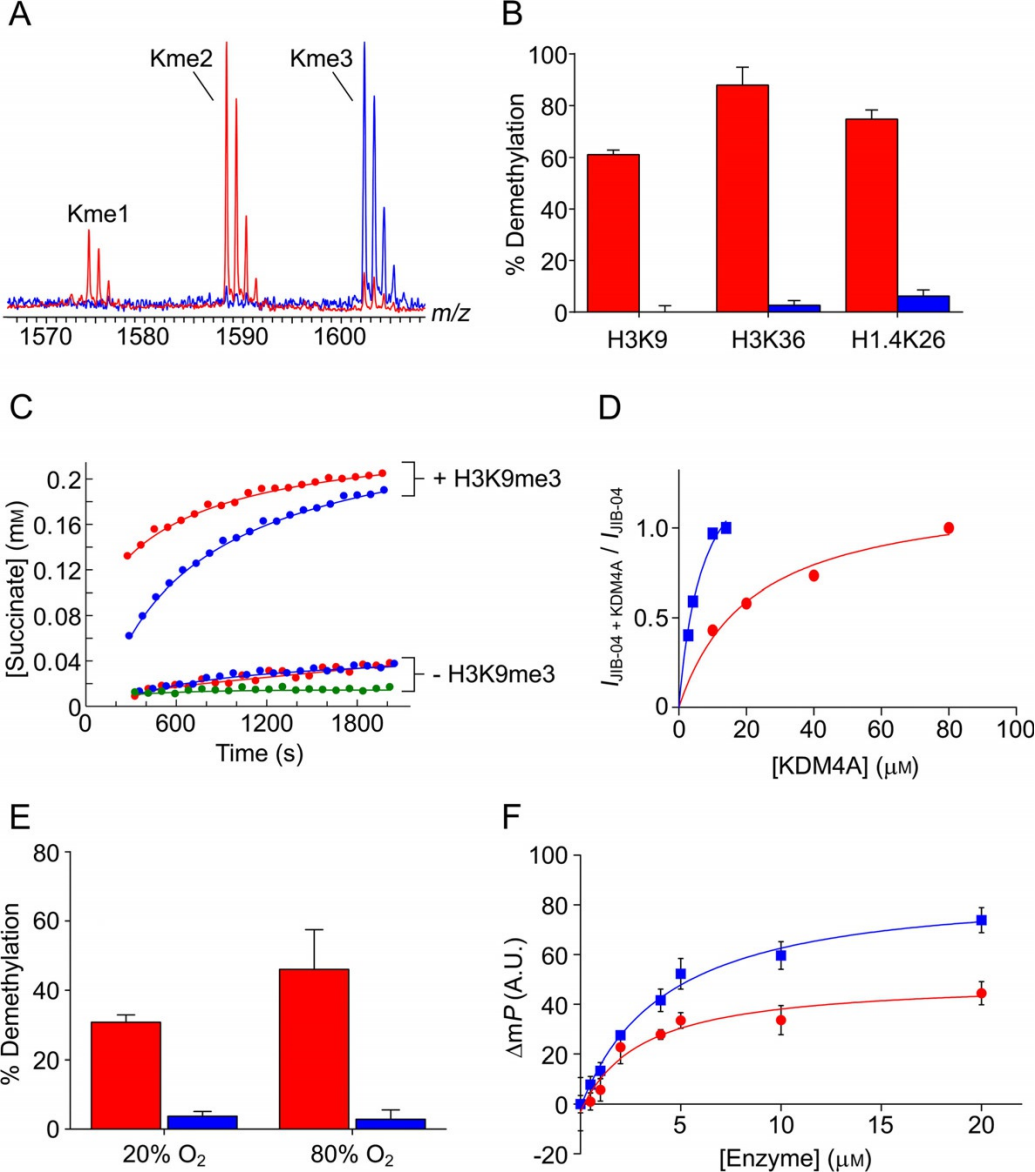
Evidence that Lys241 in KDM4A is involved in catalysis but not O_2_ binding. A) MALDI‐TOF MS spectra showing KDM4A‐catalysed demethylation of H3K9me3 peptide (ARTKQTARKme3STGGKA; 40 min; 37 °C). Clear demethylation is observed with WT KDM4A (red); however, only trace levels of dimethylated product are apparent in the sample with K241A KDM4A (blue). B) The percentage demethylation of trimethylated H3K9 peptide (sequence: ARTKQTARKme3STGGKA), H3K36 peptide (SAPATGGVKme3KPHRYR) and H1.4K26 peptide (TPVKKKARKme3SAGAAK) catalysed by WT and K241A KDM4A. Only low‐level demethylation was observed with the K241A variant. C) Succinate formation in samples of KDM4A (WT or K241A, 20.5 μm), 2OG (2 mm), ascorbate (1 mm), and ferrous iron (100 μm) in 50 mm ammonium formate buffer pH 7.5 over time, in the presence or absence of ARKme3STGGK; no‐enzyme control: green. Succinate formation rates in the absence of peptide are near identical for WT and K241A KDM4A. Succinate formation is stimulated by H3K9me3 peptide, but more readily with WT KDM4A. D) Binding curves showing binding of the KDM4‐selective inhibitor JIB‐04 to WT and K241A KDM4A. JIB‐04 is proposed to interact with K241 in the KDM4 active site; however, binding to K241A appears stronger. E) Percentage demethylation of H3K9me3 peptide catalysed by WT and K241A KDM4A at 20 and 80 % (*v*/*v*) O_2_ after 10 min at 37 °C. Demethylation increases with increased O_2_ concentration with WT KDM4A; however, the extent of demethylation with K241A KDM4A was very low (<5 %). F) The binding of a fluorescence‐labelled histone fragment (ARTKQTARKme3STGGKA‐fluorescein) is similar for WT and K241A KDM4A, respectively.

Given the role of K241 in O_2_ binding/recruitment proposed by Chen et al.,^13^ we postulated that the lack of KDM activity might be due to reduced binding of O_2_. ^1^H NMR analyses were therefore carried out to assess the ability of the WT and K241A KDM4A to catalyse substrate‐uncoupled turnover of 2OG to succinate, a process that requires O_2_ binding. Samples containing either WT or K241A (20.5 μm), an excess of 2OG (2 mm), ascorbate (1 mm), and ferrous iron (100 μm) in ammonium formate buffer (50 mm, pH 7.5) were subjected to ^1^H NMR analysis at 1.5 min intervals over a 30‐min reaction. With both WT and K241A KDM4A, the triplet ^1^H resonance at *δ*_H_=2.4 ppm (corresponding to 2OG) decreased in intensity during the analysis, while a new singlet ^1^H resonance at *δ*_H_=2.3 ppm, corresponding to succinate emerged (Figures 1 C and S3). The initial succinate formation rates were near identical with both enzymes (WT=1.35 μm min^−1^, K241A=1.55 μm min^−1^, Figure 1 C, note: 2OG turnover was too slow for accurate determination of the Michaelis–Menten kinetic parameters). Uncoupled 2OG turnover by the WT and K241A enzymes was inhibited by the 2OG competitor *N*‐oxalylglycine; interestingly, inhibition was more pronounced with the KDM4‐selective inhibitor JIB‐04 (Figures S4 and S5),^14^ which is proposed to interact with K241 in the KDM4A active site (inferred from modelling studies).^15^ Results from NMR binding experiments support preferential binding of JIB‐04 to the K241A variant over the WT enzyme (provisional *K*_D(JIB‐04)_=22±5 and 7±2 μm for WT and K241A, respectively, Figures 1 D and S6), thus implying no/suboptimal interactions between JIB‐04 and K241. Experiments were then conducted in the presence of a histone H3 eight‐residue fragment peptide containing *N* ^*ϵ*^‐trimethyllysine at position 9 (H3K9me3, sequence: ARKme3STGGK). Initial succinate formation rates were markedly increased for both the WT and K241A enzymes; this implied binding of the histone fragment, which is reported to stimulate succinate formation by KDM4,^16^ and potentially demethylation. The ^1^H resonances corresponding to lysyl methyl groups were obscured by residual glycerol and HEPES from the enzyme stocks; this hindered the detection of KDM activity. Stimulation of succinate formation was greater for WT than for K241A KDM4A.

MS studies were then carried out to investigate whether demethylation by K241A KDM4A is stimulated by increasing the O_2_ concentration. KDM assays (using the same final concentrations of components as above) were carried out with the use of a Mass Flow Controller (Brooks Instruments) to equilibrate the reactions at either 20 or 80 % O_2_, as described;12b reactions were left for 10 min before quenching (with methanol) and MALDI‐TOF MS analysis. As reported, the KDM activity of WT KDM4A increased with increasing O_2_ concentration (from 31 to 46 % demethylation, Figure 1 E).12b However, only trace demethylation (<5 %) was observed in the samples with K241A KDM4A at either 20 or 80 % oxygen (Figure 1 E); this indicated that oxygen binding/recruitment does not limiting the KDM activity of the K241A variant.

The crystal structures of KDM4A appear to show that K241 interacts with the H3 fragment peptide substrate (Scheme 1, inset);^17^ we therefore investigated whether the KDM activity of the K241A variant is limited by weakened substrate binding. The binding of WT and K241A KDM4A to a histone fragment peptide was evaluated in a fluorescence polarisation assay. A 15‐residue H3K9me3 peptide C‐terminally labelled with a fluorescein fluorophore, that is, ARTKQTARKme3STGGKA‐fluorescein, was found by MALDI‐TOF MS to be a WT KDM4A demethylation substrate (a mass shift of −14 Da was observed under standard conditions). The labelled substrate (20 nm) was incubated with the 2OG analogue *N*‐oxalylglycine (1 mm),^18^ NiCl_2_ (10 μm) and various concentrations of the WT and K241A enzymes (0–20 μm). The fluorescence emission spectra of the samples parallel and perpendicular to the excitation plane were then measured, and used to calculate the fluorescence polarisation at each enzyme concentration. The apparent binding constants for peptide binding were near identical for both enzymes (3.06±2.9 μm for WT KDM4A, and 4.54±2.1 μm for K241A KDM4A, respectively), thus indicating that mutation of the K241 residue to alanine does not alter the affinity of KDM4A for the peptide substrate (Figure 1 F).

The overall MS, NMR spectroscopy and fluorescence polarisation studies imply that K241 has a role in KDM4A catalysis. However, the observation that the K241A variant catalyses uncoupled succinate formation with comparable efficiency to the WT implies that the role(s) of K241 during catalysis do not involve (at least initial) binding of O_2_.^13^ Thus, the observed mixed‐mode inhibition^14^ of K241A by JIB‐04 probably does not reflect direct disruption of O_2_ binding. Succinate formation by the K241A variant is stimulated by a histone fragment peptide, and a fluorescently labelled histone peptide binds in a similarly tight manner to the WT and K241A KDM4A. Thus, K241 appears to have a role in coupling 2OG turnover to substrate oxidation. One possibility is that K241 helps to orientate the methylated lysyl side chain within the KDM4A active site, thereby ensuring efficient oxidation of a methyl C−H bond after succinate formation. K241 might also limit the access to the active site of water molecules that could quench the reactive iron(IV)‐oxo intermediate before methyl C−H bond oxidation can occur. Targeting K241 is of interest both with respect to developing classical tightly binding inhibitors and to developing inhibitors that decouple 2OG and substrate oxidation. Such inhibitors are of interest because uncoupling could cause inactivation; in the absence of “prime” substrate, self‐oxidation of 2OG oxygenases can occur, either directly or through the production of reactive oxygen species.^19^ Therefore, molecules that bind and induce self‐oxidation before dissociating, for example, K241 binders, have the potential to be catalytic inactivators.

## Conflict of interest

*The authors declare no conflict of interest*.

## Supporting information

As a service to our authors and readers, this journal provides supporting information supplied by the authors. Such materials are peer reviewed and may be re‐organized for online delivery, but are not copy‐edited or typeset. Technical support issues arising from supporting information (other than missing files) should be addressed to the authors.

Supplementary

## References

[1] X. Cheng , R. C. Trievel in 2-Oxoglutarate-Dependent Oxygenases, The Royal Society of Chemistry, 2015, pp. 210–245.

[2a] Y. Shi , F. Lan , C. Matson , P. Mulligan , J. R. Whetstine , P. A. Cole , R. A. Casero , Y. Shi , Cell 2004, 119, 941–953; 15620353 10.1016/j.cell.2004.12.012

[2b] D. N. Ciccone , H. Su , S. Hevi , F. Gay , H. Lei , J. Bajko , G. Xu , E. Li , T. Chen , Nature 2009, 461, 415–418. 19727073 10.1038/nature08315

[3a] Y. Tsukada , J. Fang , H. Erdjument-Bromage , M. E. Warren , C. H. Borchers , P. Tempst , Y. Zhang , Nature 2006, 439, 811–816; 16362057 10.1038/nature04433

[3b] J. R. Whetstine , A. Nottke , F. Lan , M. Huarte , S. Smolikov , Z. Chen , E. Spooner , E. Li , G. Zhang , M. Colaiacovo , Y. Shi , Cell 2006, 125, 467–481; 16603238 10.1016/j.cell.2006.03.028

[3c] R. J. Klose , K. Yamane , Y. Bae , D. Zhang , H. Erdjument-Bromage , P. Tempst , J. Wong , Y. Zhang , Nature 2006, 442, 312–316; 16732292 10.1038/nature04853

[3d] K. Yamane , C. Toumazou , Y. Tsukada , H. Erdjument-Bromage , P. Tempst , J. Wong , Y. Zhang , Cell 2006, 125, 483–495; 16603237 10.1016/j.cell.2006.03.027

[3e] R. J. Klose , Q. Yan , Z. Tothova , K. Yamane , H. Erdjument-Bromage , P. Tempst , D. G. Gilliland , Y. Zhang , W. G. Kaelin, Jr. , Cell 2007, 128, 889–900; 17320163 10.1016/j.cell.2007.02.013

[3f] W. Feng , M. Yonezawa , Y. Jung , T. Jenuwein , I. Grummt , Nat. Struct. Mol. Biol. 2010, 17, 445–450; 20208542 10.1038/nsmb.1778

[3g] C. Loenarz , W. Ge , M. L. Coleman , N. R. Rose , C. D. Cooper , R. J. Klose , P. J. Ratcliffe , C. J. Schofield , Hum. Mol. Genet. 2010, 19, 217–222. 19843542 10.1093/hmg/ddp480 PMC4673897

[4] L. J. Walport , R. J. Hopkinson , C. J. Schofield , Curr. Opin. Chem. Biol. 2012, 16, 525–534. 23063108 10.1016/j.cbpa.2012.09.015

[5] S. M. Kooistra , K. Helin , Nat. Rev. Mol. Cell Biol. 2012, 13, 297–311. 22473470 10.1038/nrm3327

[6a] S. T. Williams , L. J. Walport , R. J. Hopkinson , S. K. Madden , R. Chowdhury , C. J. Schofield , A. Kawamura , Epigenetics 2014, 9, 1596–1603; 25625844 10.4161/15592294.2014.983381 PMC4623018

[6b] P. Trojer , J. Zhang , M. Yonezawa , A. Schmidt , H. Zheng , T. Jenuwein , D. Reinberg , J. Biol. Chem. 2009, 284, 8395–8405. 19144645 10.1074/jbc.M807818200 PMC2659197

[7] L. J. Walport , R. J. Hopkinson , R. Chowdhury , R. Schiller , W. Ge , A. Kawamura , C. J. Schofield , Nat. Commun. 2016, 7, 11974. 27337104 10.1038/ncomms11974 PMC4931022

[8] J. M. LaSalle , OA Autism 2013, 1, 14. 24404383 10.13172/2052-7810-1-2-610 PMC3882126

[9] Q.-J. Zhang , H.-Z. Chen , L. Wang , D.-P. Liu , J. A. Hill , Z.-P. Liu , J. Clin. Invest. 2011, 121, 2447–2456. 21555854 10.1172/JCI46277 PMC3104772

[10a] M. Tahiliani , P. Mei , R. Fang , T. Leonor , M. Rutenberg , F. Shimizu , J. Li , A. Rao , Y. Shi , Nature 2007, 447, 601; 17468742 10.1038/nature05823

[10b] H. H. Qi , M. Sarkissian , G.-Q. Hu , Z. Wang , A. Bhattacharjee , D. B. Gordon , M. Gonzales , F. Lan , P. P. Ongusaha , M. Huarte , N. K. Yaghi , H. Lim , B. A. Garcia , L. Brizuela , K. Zhao , T. M. Roberts , Y. Shi , Nature 2010, 466, 503–507. 20622853 10.1038/nature09261 PMC3072215

[11] J. W. Højfeldt , K. Agger , K. Helin , Nat. Rev. Drug Discovery 2013, 12, 917–930. 24232376 10.1038/nrd4154

[12a] E. M. Sánchez-Fernández , H. Tarhonskaya , K. Al-Qahtani , R. J. Hopkinson , J. S. McCullagh , C. J. Schofield , E. Flashman , Biochem. J. 2013, 449, 491–496; 23092293 10.1042/BJ20121155 PMC4673901

[12b] R. L. Hancock , N. Masson , K. Dunne , E. Flashman , A. Kawamura , ACS Chem. Biol. 2017, 12, 1011–1019. 28051298 10.1021/acschembio.6b00958 PMC5404277

[13] Z. Chen , J. Zang , J. Kappler , X. Hong , F. Crawford , Q. Wang , F. Lan , C. Jiang , J. Whetstine , S. Dai , K. Hansen , Y. Shi , G. Zhang , Proc. Natl. Acad. Sci. USA 2007, 104, 10818–10823. 17567753 10.1073/pnas.0704525104 PMC1891149

[14] L. Wang , J. Chang , D. Varghese , M. Dellinger , S. Kumar , A. M. Best , J. Ruiz , R. Bruick , S. Peña-Llopis , J. Xu , D. J. Babinski , D. E. Frantz , R. A. Brekken , A. M. Quinn , A. Simeonov , J. Easmon , E. D. Martinez , Nat. Commun. 2013, 4, 2035. 23792809 10.1038/ncomms3035 PMC3724450

[15] B. Cascella , S. G. Lee , S. Singh , J. M. Jez , L. M. Mirica , Chem. Commun. 2017, 53, 2174–2177. 10.1039/c6cc09882g PMC5511625 28144654

[16a] R. J. Hopkinson , R. B. Hamed , N. R. Rose , T. D. Claridge , C. J. Schofield , ChemBioChem 2010, 11, 506–510; 20095001 10.1002/cbic.200900713

[16b] R. J. Hopkinson , L. J. Walport , M. Münzel , N. R. Rose , T. J. Smart , A. Kawamura , T. D. W. Claridge , C. J. Schofield , Angew. Chem. Int. Ed. 2013, 52, 7709–7713; 10.1002/anie.201303282 PMC3798130 23788451

[16c] L. J. Walport , R. J. Hopkinson , M. Vollmar , S. K. Madden , C. Gileadi , U. Oppermann , C. J. Schofield , C. Johansson , J. Biol. Chem. 2014, 289, 18302–18313. 24798337 10.1074/jbc.M114.555052 PMC4140284

[17] S. S. Ng , K. L. Kavanagh , M. A. McDonough , D. Butler , E. S. Pilka , B. M. R. Lienard , J. E. Bray , P. Savitsky , O. Gileadi , F. von Delft , N. R. Rose , J. Offer , J. C. Scheinost , T. Borowski , M. Sundstrom , C. J. Schofield , U. Oppermann , Nature 2007, 448, 87–91. 17589501 10.1038/nature05971

[18] R. J. Hopkinson , A. Tumber , C. Yapp , R. Chowdhury , W. Aik , K. H. Che , X. S. Li , J. B. L. Kristensen , O. N. F. King , M. C. Chan , K. K. Yeoh , H. Choi , L. J. Walport , C. C. Thinnes , J. T. Bush , C. Lejeune , A. M. Rydzik , N. R. Rose , E. A. Bagg , M. A. McDonough , T. J. Krojer , W. W. Yue , S. S. Ng , L. Olsen , P. E. Brennan , U. Oppermann , S. Müller , R. J. Klose , P. J. Ratcliffe , C. J. Schofield , A. Kawamura , Chem. Sci. 2013, 4, 3110–3117. 26682036 10.1039/C3SC51122G PMC4678600

[19] M. Mantri , Z. Zhang , M. A. McDonough , C. J. Schofield , FEBS J. 2012, 279, 1563–1575. 22251775 10.1111/j.1742-4658.2012.08496.x

