# Supplementary material for: Lysine‐241 Has a Role in Coupling 2OG Turnover with Substrate Oxidation During KDM4‐Catalysed Histone Demethylation
Source: Chembiochem. 2018 Apr 6;19(9):917–21. doi: 10.1002/cbic.201800002 (PMC5969224; doi:10.1002/cbic.201800002)
Supplement: Supplementary file 1 — Supplementary [file CBIC-19-917-s001.pdf]

CHEMBIOCHEM

Supporting Information

Lysine-241 Has a Role in Coupling 2OG Turnover with Substrate Oxidation During KDM4-Catalysed Histone Demethylation

Rebecca L. Hancock,[a] Martine I. Abboud+,[a] Tristan J. Smart+,[a] Emily Flashman,[a] Akane Kawamura,[a] Christopher J. Schofield,*[a] and Richard J. Hopkinson*[a, b]

cbic_201800002_sm_miscellaneous_information.pdf

EXPERIMENTAL SECTION

Production of recombinant KDM4A WT and K241A: N-Terminally His_6-tagged truncated WT KDM4A1-359 and KDM4A1-359 K241A were recombinantly produced in *Escherichia coli* BL21(DE3) cells and purified by Ni-affinity and size-exclusion chromatography, as previously described.[1] Protein solutions were stored in 50 mM HEPES, with 500 mM NaCl, 5 % (*v/v*) glycerol, pH 7.5, at −80 °C.

MALDI-TOF MS activity assays: 1 µM WT KDM4A or KDM4A K241A was incubated with 100 µM disodium 2-oxoglutarate (2OG), 100 µM sodium L-ascorbate, 10 µM ferrous iron (source $(NH_4)_2FeSO_4.6H_2O$) and 100 µM of 15-residue H3 fragment peptide containing the requisite trimethyllysine mark (Table S1) in 50 mM HEPES buffer pH 7.5 for 40 min at 37 °C. O_2 dependence assays were performed using the above reaction conditions, as described previously.[2] Briefly, a Mass Flow controller (Brooks Instruments) was used to equilibrate a mixture of H3 1-15 K9me3 peptide and 50 mM HEPES buffer pH 7.5 to the required O_2 concentration (20 or 80 %) at 37 °C for 10 min. After addition of L-ascorbate (1 µL), 2OG (1 µL) and ferrous iron (2 µL), reactions were initiated by the addition of either WT KDM4A or KDM4A K241A (1 µL) and incubated for 10 min. Assays were quenched with an equal volume of methanol, and the extent of the reaction determined using MALDI-TOF mass spectrometry (MS). Data were analysed using MASSLYNX 4.0 (Waters).

^{1}H NMR activity and inhibition assays: 20.5 µM KDM4A WT or KDM4A K241A was mixed with 2 mM 2OG, 1 mM sodium L-ascorbate, and 100 µM ferrous iron (source $(NH_4)_2FeSO_4.6H_2O$), in 50 mM ammonium formate buffer pH 7.5 10 % *v/v* D_2O in an Eppendorf tube, before being immediately transferred to an NMR tube and subjected to ^{1}H NMR analysis (700 MHz). For samples containing peptide substrate (sequence: ARKme3STGGK), the peptide concentration was 300 µM. For inhibition assays, samples contained 100 µM inhibitor (JIB-04 or NOG) and the spectra were acquired after 35 min. Note: residual protein buffer was also present. NMR analyses were carried out on a Bruker AVIII 700 spectrometer equipped with an inverse TCI ^{1}H/^{13}C/^{15}N cryoprobe and running TOPSPIN 3.2 software. The deuterium signal was used as an internal lock signal and 1H chemical shifts are reported in ppm relative to the solvent HDO resonance (δ_H 4.7 ppm). The solvent signal was reduced by excitation sculpting.[3]

^{1}H CPMG NMR binding assays: Typical experimental parameters for Carr-Purcell-Meiboom-Gill (CPMG) NMR spectroscopy were as follows: total echo time, 40 ms; relaxation delay, 2 s; and number of transients, 264. The PROJECT-CPMG sequence was applied

(90°x−[τ−180°y−τ−90°y−τ−180°y−τ]n−acq).[4] Water suppression was achieved by presaturation. Protein (KDM4A WT or KDM4A K241A) was titrated into an assay mixture of 40 µM JIB-04 and 100 µM ferrous iron (source $(NH_4)_2FeSO_4.6H_2O$), in 50 mM ammonium formate buffer, pH 7.5, 10 % *v/v* D_2O in 3 mm NMR MATCH tube and subjected to ^{1}H CPMG NMR analysis (600 MHz). K_D was fitted by using the quadratic equation as previously reported.[5]

Fluorescence polarisation: WT KDM4A and KDM4A K241A were each titrated (0−20 µM) against 20 nM of a 15-residue H3K9me3 peptide C-terminally labelled with a fluorescein fluorophore (sequence: ARTKQTARKme3STGGKA-fluorescein). Reaction mixtures also contained 10 µM $NiCl_2$ and 1 mM *N*-oxalylglycine, and were incubated at room temperature for 10 min before analysis. Assays were performed in 384 well, PS, µClear® black, non-binding plates (Greiner BioOne) in a PHERAstar FS® microplate reader (BMG Labtech), with fluorescence excitation at 485 nm and fluorescence emission measured both perpendicular and parallel to the excitation plane at 520 nm. Polarisation (mP) was evaluated using Mars v2.41 machine (BMG Labtech), and data were fitted to a one-site total binding model using GraphPad Prism v5.0.

Table S1: Sequence of 15-mer peptides (all C-terminal amides) used in MALDI-TOF MS activity assays.

**Substrate**	**Sequence**
H3K9me3	ARTKQTARKme3STGGKA
H3K36me3	SAPATGGVKme3KPHRYR
H1.4K26me3	TPVKKKARKme3SAGAAK

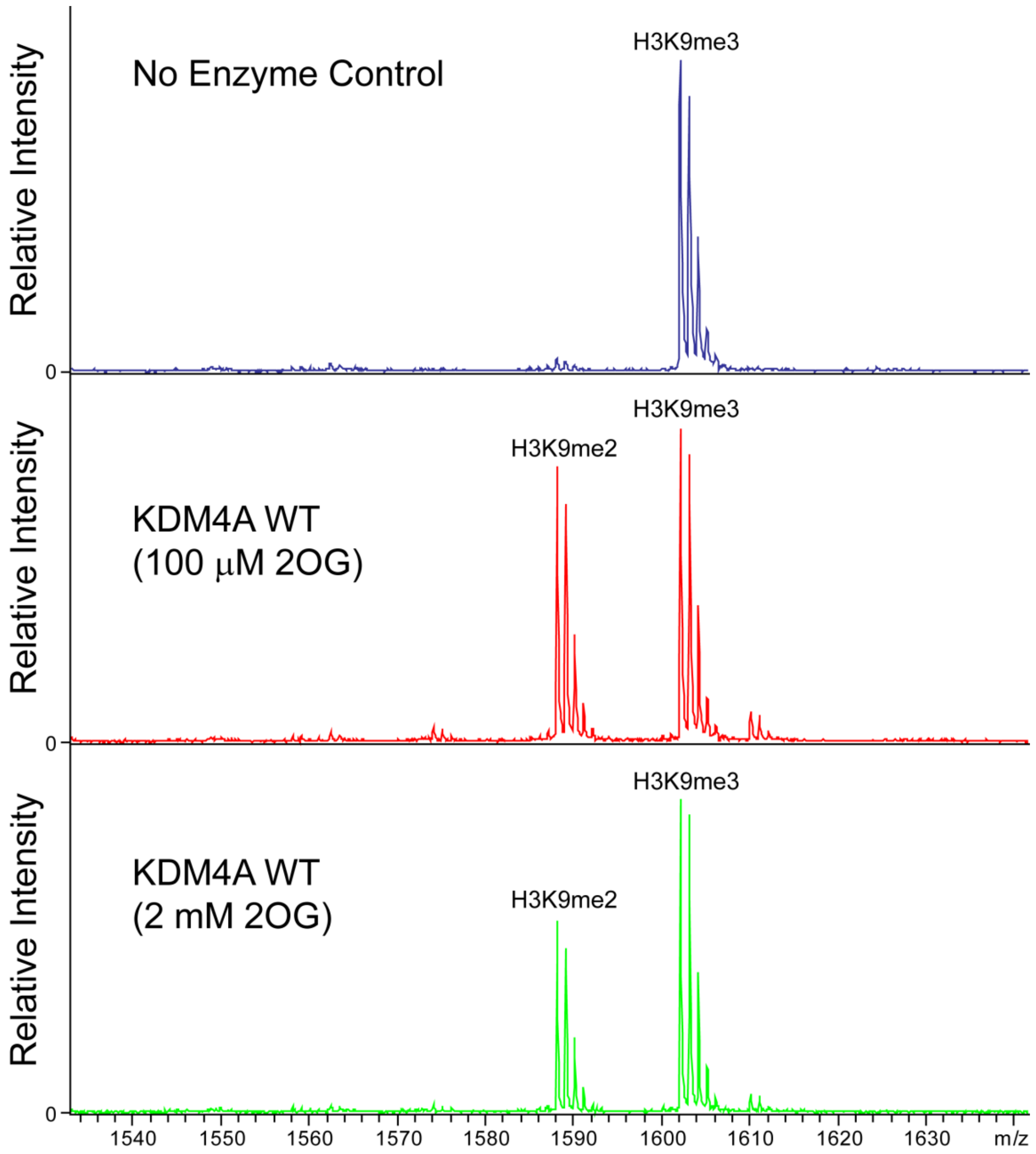

Figure S1: MALDI-MS spectra showing KDM4A WT-catalysed demethylation of H3K9me3 15mer peptide after 40 minutes at 37 °C. Demethylation was marginally less efficient at 2 mM 2OG.

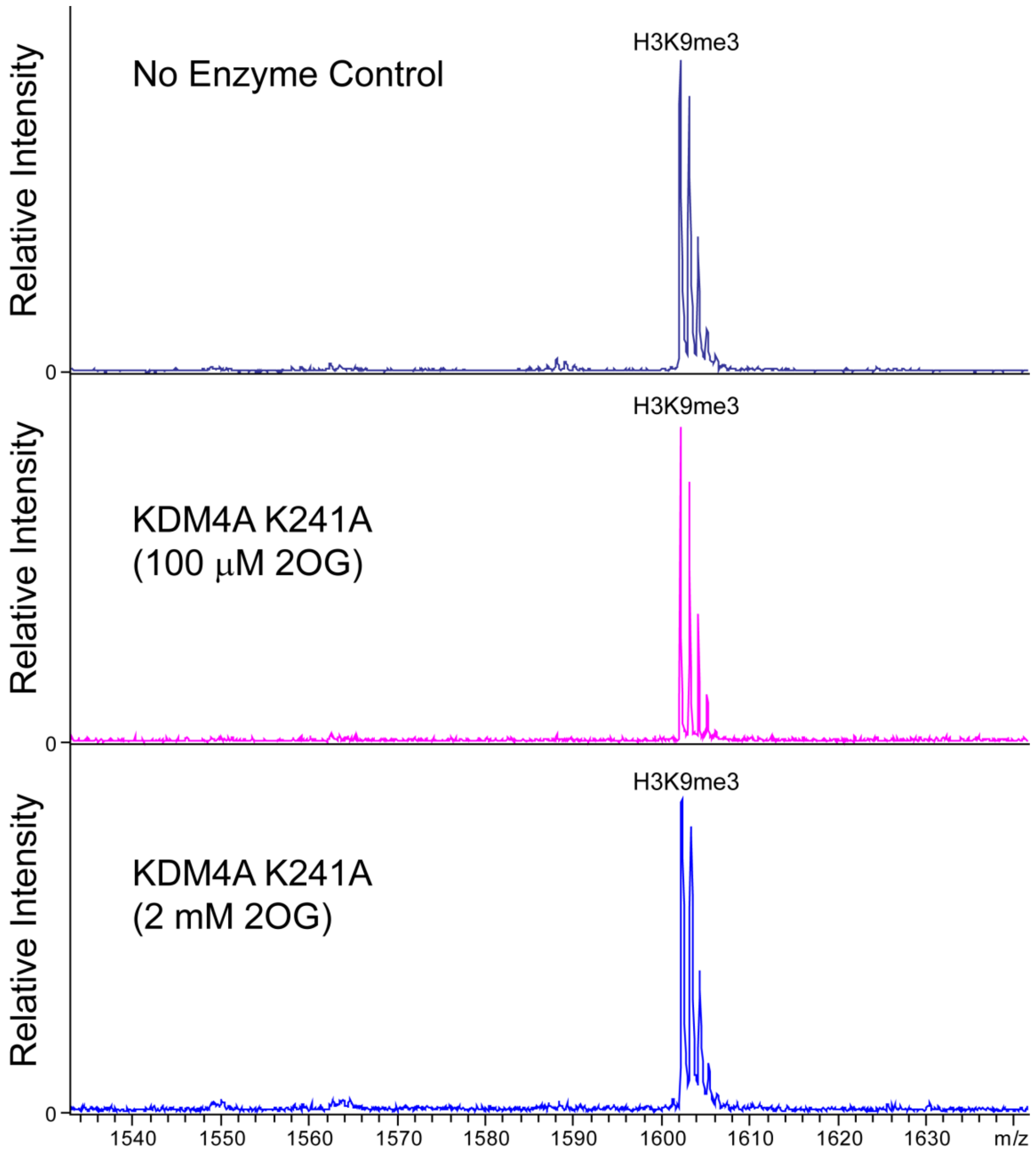

Figure S2: MALDI-MS spectra showing KDM4A K241A-catalysed demethylation of H3K9me3 15mer peptide after 40 minutes at 37 °C. No/minimal demethylation was observed at either 100 µM or 2 mM 2OG concentrations.

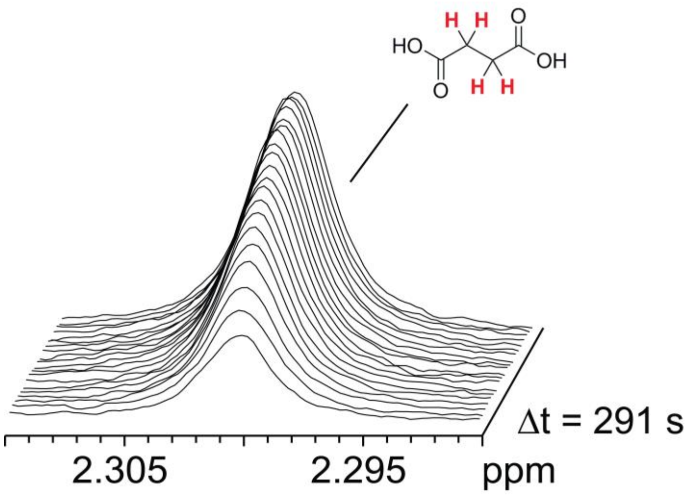

Figure S3: ^{1}H NMR monitoring of a sample containing KDM4A K241A (20.5 μM), 2OG (2 mM), ascorbate (1 mM), and ferrous iron (100 μM) in 50 mM ammonium formate buffer pH 7.5. The emergence of a singlet (δ_H 2.3 ppm) indicates succinate formation.

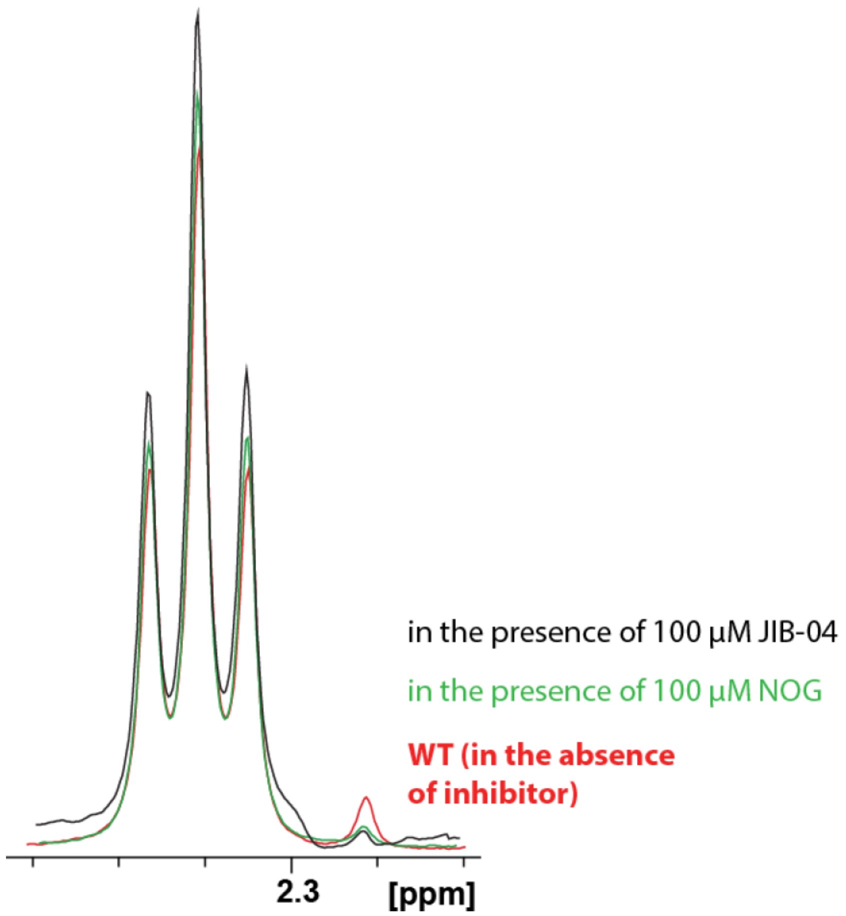

Figure S4: ^{1}H NMR spectra showing inhibition of KDM4A K241A-catalysed uncoupled 2OG turnover by *N*-oxalylglycine (NOG, green) and JIB-04 (black). Conversion of 2OG (triplet at δ_H 2.31 ppm) to succinate (singlet at δ_H 2.29 ppm) is decreased in the presence of NOG and JIB-04.

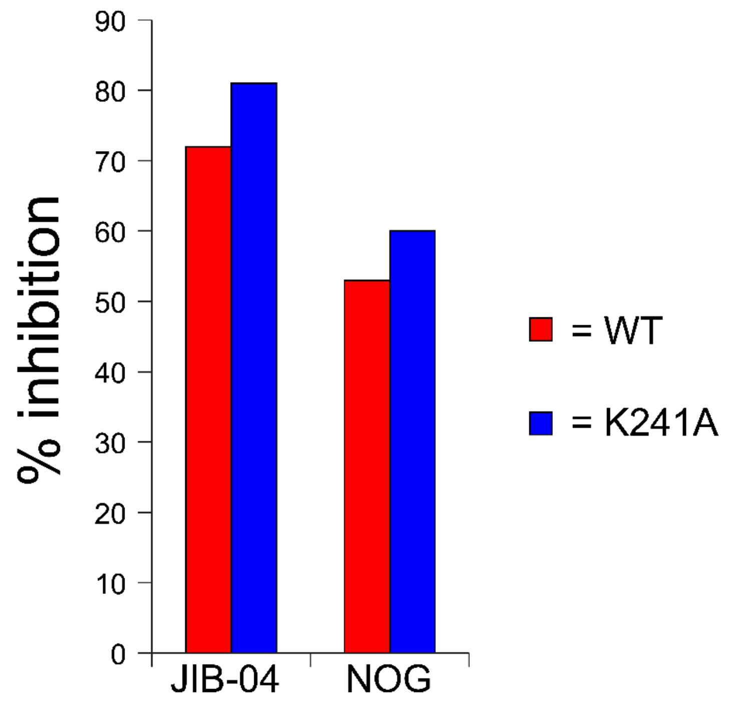

Figure S5: Graph showing percentage inhibition of KDM4A-catalysed conversion of 2OG to succinate (uncoupled) in the presence of inhibitors (at 100 μM) relative to DMSO control. Inhibition was similar with the WT (red) and the K241A variant (blue) enzymes.

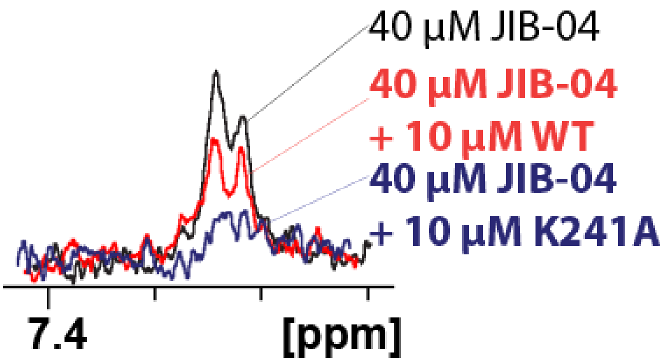

Figure S6: ^{1}H PROJECT-CPMG spectra showing binding of JIB-04 to KDM4A WT (red) and KDM4A K241A (blue), Decreased signal intensity indicates binding of JIB-04 to the enzyme. Binding appears stronger to the K241 variant than KDM4A WT.

References

[1] S. S. Ng, K. L. Kavanagh, M. A. McDonough, D. Butler, E. S. Pilka, B. M. Lienard, J. E. Bray, P. Savitsky, C. Gileadi, F. Von Delft, N. R. Rose, J. Offer, J. C. Scheinost, T. Borowski, M. Sundstrom, C. J. Schofield, U. Oppermann, *Nature* **2007**, *448*, 87-91.

[2] R. L. Hancock, N. Masson, K. Dunne, E. Flashman, A. Kawamura, *ACS Chemical Biology* **2017**, *12*, 1011-1019.

[3] T. L. Hwang, A. J. Shaka, *Journal of Magnetic Resonance, Series A* **1995**, *112*, 275-279.

[4] J. A. Aguilar, M. Nilsson, G. Bodenhausen, G. A. Morris, *Chemical Communications* **2012**, *48*, 811-813.

[5] I. K. Leung, M. Demetriades, A. P. Hardy, C. Lejeune, T. J. Smart, A. Szollossi, A. Kawamura, C. J. Schofield, T. D. Claridge, *J. Med. Chem.* **2013**, *56*, 547-555.
